# Evaluation of the Association between Androgen Receptor and AURKA and Its Prognostic Value in Gastric Cancer

**Published:** 2019-10-01

**Authors:** Shahrzad Soleymani Fard, Masoud Sotoudeh, Mansour Yazdanbod, Ardeshir Ghavamzadeh, Reza Malekzadeh, Marjan Yaghmaie, Seyed Asadollah Mousavi, Seyed H. Ghaffari, Kamran Alimoghaddam

**Affiliations:** 1 Hematology, Oncology and Stem Cell Transplantation Research Institute, Tehran University of Medical Sciences, Tehran, Iran; 2 Digestive Oncology Research Center, Digestive Diseases Research Institute, Shariati Hospital, Tehran University of Medical Sciences, Tehran, Iran; 3 Department of Surgery, Madaen Hospital, Tehran, Iran

**Keywords:** Aurora kinase A (AURKA), Androgen receptor (AR), Gastric cancer (GC), Prognostic marker

## Abstract

**Background:** It is well-known that Aurora kinase A (AURKA) shows oncogenic properties in various tumor types including gastric cancer (GC). Moreover, previous studies have demonstrated that AURKA has a specific androgen receptor (AR) binding site in its promoter; thus, it could be regulated by AR. Since it has been shown that AR overexpresses in gastric cancer (GC) as a male-predominant tumor, the goal of this study was to evaluate the association between AR and AURKA and its prognostic value in GC patients.

**Materials and Methods**: We assessed the expression profile of AURKA in 60 fresh GC and adjacent non-tumor tissues and 50 normal gastric specimen by qRT-PCR, and investigated the association of AURKA expression with clinicopathological features. Furthermore, we evaluated possible correlation between AURKA and AR to elucidate a novel prognostic marker using Kaplan-Meier method and Cox regression model.

**Results:** Among GC patients, 65% (39/60) overexpressed AURKA relative to normal gastric tissues. AURKA overexpression was significantly correlated with the AR overexpression in GC patients. Although AURKA expression alone was not remarkably associated with poor outcome, we provided some evidence that combined evaluation of AURKA and AR expression could independently predict survival of GC patients adjusted for other variables (HR=1.7, CI=1.314-3.833 p=0.042).

**Conclusion: **These results indicate that AR and AURKA may crosstalk to promote GC progression. Our findings have clinical importance because they suggest simultaneous assessment of AURKA and AR expression as a novel potential prognostic marker.

## Introduction

 Gastric cancer (GC), the third leading cause of cancer-related death worldwide, is extremely aggressive and invasive ^1^. Mounting studies have revealed the association of various genes with GC progression. However, the exact mechanisms

underlying the progression and development of GC are not fully elucidated. Therefore, the molecular factors responsible for aggressiveness of GC should be assessed profoundly. 

Accurate mitotic processing needs main mitotic kinases. One particular mitotic kinase is Aurora kinase A (AURKA) which is a serine-threonine kinase and functions in mitotic spindle formation ^2^^, ^^3^. Previous studies have demonstrated that aberrant expression of AURKA shows oncogenic properties and could cause centrosome amplification, cytokinesis failure, and subsequently aneuploidy ^4^^, ^^5^. Amplification and overexpression of AURKA is frequently reported in gastrointestinal cancers ^6^^-^^8^.

Androgen receptor, which is a member of nuclear receptor superfamily acts as a transcription factor that regulates the expression of several genes ^9^. AR could act as an oncoprotein and modulate metastasis and progression of several cancer types when aberrantly expressed ^10^^, ^^11^. Recently, some studies have indicated the role of AR in gastric cancer as a male-predominant tumor ^12^^, ^^13^. 

There are several studies demonstrating interaction between AURKA and AR in prostate cancer ^14^^, ^^15^. They have shown that in LNCaP cells expressing high levels of AR, androgen stimulation could increase level of AURKA expression. In a recent study, Kivinummi, et al. revealed that in AR-positive CRPC samples which carried amplification of AR gene and/or expressed AR in high levels, AURKA was significantly overexpressed ^16^.

Therefore, the aim of this study was to investigate the potential association between AURKA and AR genes expressions in GC patients for the first time. Moreover, we assessed the AURKA expression in GC tissues, adjacent non-tumor tissues and normal gastric samples and evaluated its correlation with clinicopathological characteristics. Finally, we asked if the correlation between AURKA and AR genes could introduce a novel prognostic marker for GC patients.

## MATERIALS AND METHODS

**Patients and clinicopathological data**

In the present cohort study, 60 fresh tissue samples were collected from gastric cancer patients who underwent surgical resection at Madaen, Kasra or Imam Khomeini hospital, Tehran, Iran, between June 2016 and June 2017. All patients were pathologically and clinically diagnosed with GC; moreover, patients who received chemotherapy or radiotherapy before surgery or patients with double primary tumors were excluded. Fresh tumor tissue specimens and adjacent non-tumor tissues were prepared within 15 min of excision, stabilized in RNAlater solution (RNAlater RNA Stabilization Reagent, QIAGEN, Germany) at 4°C overnight and preserved at −20°C until RNA extraction. The patients were followed up until death or the end of the study (September, 2018). Overall survival (O.S) refers to the time (months) between the date of surgery and the date of death or at the end of follow-up.

Furthermore, 50 fresh samples were obtained from normal cases underwent endoscopy procedure in Digestive Diseases Research Institute, Shariati hospital, Tehran, Iran.

The informed consents were signed by all participating patients or their first family members. The Clinical Research Ethics Committee of Tehran University of Medical School (TUMS) approved our research. This study is complied with the ethical principles of the HORC-SCT, Shariati hospital and the Helsinki Declaration of 1964 and later versions. 

(Ethics committee approval code: ir.TUMS.horcsct.rec.1394.103.10).

**Total RNA preparation and reverse transcription PCR**

Total RNA was extracted from the RNAlater-stabilized tissues or cell line lysates using 1 ml RiboEx reagent (GeneAll Biotechnology Co, South Korea). cDNA was synthesized using PrimeScriptTM RT reagent Kit (TaKaRa, Japan). The reaction vessel was incubated in an ABI Veriti Thermocycler (Applied Biosystems) for 15 min at 37°C, and 5 second at 85°C. The control gene used in this study was human beta-2-microglobulin (B2M). 

**Real-time quantitative PCR**

A LightCycler 96 instrument (Roche Molecular Diagnostics) was applied to perform the quantitative reverse transcription-PCR (qRT-PCR) analysis using SYBRGreen RealQ-PCR Master Mix kit (Ampliqon, Copenhagen, Denmark) as described by the manufacturer. Thermal cycling condition consisted of an activation step for 15 min at 95 °C followed by 40 cycles of denaturation step (15 s at 95°C) and a combined annealing/extension step for 1 min at 60 °C. Water instead of cDNA included in the PCR reaction as negative controls. In the present study, we used two different housekeeping genes (hypoxanthine phosphoribosyl transferase1 (HPRT) and beta-2-microglobulin (B2M)) for normalization of target genes expression levels. However, B2M proved to be the more stable among the evaluated genes, and showed no variation between tissues.

mRNA expression levels were quantified as ΔCt values by comparing it with the mean Ct values of beta-2-microglobulin (B2M) taken as reference/endogenous control gene (ΔCt = Ct target- Ct reference) to normalize the possible differences in the amount of total RNA. The relative expression levels were calculated using the 2^− (ΔΔCT)^ method according to the following formula: ΔΔCT= ΔCt tumor – ΔCt normal ^17^.

The sequences of primers used in the present study are listed in Table 1.

**Table1:** Nucleotide sequences of the primers used for QRT-PCR

**Gene**	**Accession ** **number**	**Forward Primer**	**Reverse Primer**
B2M	NM_004048	GATGAGTATGCCTGCCGTGT	CTGCTTACATGTCTCGAT CCCA
HPRT	NM_000194	TGGACAGGACTGAACGTCTT G	CCAGCAGGTCAGCAAAG AATTTA
AR	NM_000044	TTGTCCATCTTGTCGTCTTCG G	GCCTCTCCTTCCTCCTGT AGT
AURKA	NM_198433	GGATATCTCAGTGGCGGACG	GCAATGGAGTGAGACCCTCT

**Statistical analysis**

Difference in expression of AURKA between gastric tumors and adjacent non-tumor tissues or normal tissues was compared by Independent samples Mann-Whitney U test. Correlation was computed using Spearman rank test. The associations between expression of AURKA and clinicopathological characteristics were evaluated using Chi-square or Fisher’s exact tests. The survival rate was analyzed by Kaplan-Meier method (log-rank test). Univariate and multivariate survival analysis was performed by the Cox proportional hazards model to evaluate the prognostic value of known categorical variables and AURKA expression. All significant factors (p <0.05) in the univariate analysis were used for multivariate evaluation. Stepwise backward elimination was used till only significant variables maintained in multivariate model. Computerized statistical analyses were performed by the IBM SPSS^®^ statistics 22 software, and a two-tailed p< 0.05 was considered statistically significant.

## Results

**Clinicopathological Characteristics.**

The present study has investigated the clinicopathological significance of AURKA expression in gastric cancer and the correlation with androgen receptor. Sixty gastric cancer patients were included in the project.

Clinicopathological characteristics of GC patients are listed in Table 2. Moreover, fifty normal cases, including 25 females and 25 males with a median age of 51 years (age range, 19-83) were also collected.

In the present study, one sample from normal cases which had the highest ΔCt value was used as a calibrator for each specific gene. All other samples from three different groups (tumor tissues, non-tumor adjacent tissues and normal tissues) were compared with the calibrator to calculate the fold change in gene expression. Next, we determined the cut off value using ROC curve (receiver operating characteristic curve) for all mentioned genes. Values higher than cut off point was considered as overexpression and the values lower than cut off point was considered as underexpression.

AURKA overexpression has been shown to have a statistically significant correlation with lymphovascular invasion, advanced TNM stages and AR gene overexpression (Table 2). Among 37 GC patients overexpressing AR, only 4 patients underexpressed AURKA and 34 patients overexpressed AURKA (*p*<0.001). No remarkable association was found between AURKA expression and age or gender. This analysis was based on comparing tumor tissues which showed increased AURKA expression with normal gastric tissues.

**Table 2:** Association between AURKA expression and clinicopathological characteristics of patients with gastric cancer

Clinical variables	Total patients: n (%)	Evaluable patients: n (%)		
	60 (100)	Overexpressed No. (%)	Underexpressed No. (%)	*P*
**Age (years) median, range** **➢** ** n < 63** **➢** ** n ≥ 63**	63, 33-83 29 (48.3) 31 (51.7)	16 (26.7) 23 (38.3)	13 (21.7) 8 (13.3)	0.123
**Sex** **➢** ** male** **➢** ** female**	39 (65) 21 (35)	25 (41.7) 14 (23.3)	14 (23.3) 7 (11.7)	0.843
**Tumor size (cm)** **➢** ** n <5** **➢** ** n ≥ 5**	17 (28.3) 43 (71.7)	9 (15) 30 (50)	8 (13.3) 13 (21.7)	0.218
**Lauren’s classification ** **➢** ** Intestinal** **➢** ** Diffuse**	55 (91.7) 5 (8.3)	35 (58.3) 4 (6.7)	20 (33.3) 1 (1.7)	0.649
**Tumor grade** **➢** ** poorly differentiated** **➢** ** moderately differentiated** **➢** ** well differentiated**	33 (55) 21 (35) 6 (10)	21 (35) 15 (25) 3 (5)	12 (20) 6 (10) 3 (5)	0.578
**Tumor type** **➢** ** Adenocarcinoma** **➢** ** signet ring cell carcinoma**	46 (76.7) 14 (23.3)	28 (46.7) 11 (18.3)	18 (30) 3 (5)	0.224
**Lymphovascular invasion** **➢** ** YES** **➢** ** NO**	43 (71.7) 17 (28.3)	31 (51.7) 8 (13.3)	12 (20) 9 (15)	0.048
**perineural invasion** **➢** ** YES** **➢** ** NO**	50 (83.3) 10 (16.7)	33 (55) 6 (10)	17 (28.3) 4 (6.7)	1.000
**Tumor shape** **➢** ** Ulcerated flat** **➢** ** Linitis plastica** **➢** ** Polypoid**	48 (80) 5 (8.3) 7 (11.7)	31 (51.7) 4 (6.7) 4 (6.7)	17 (28.3) 1 (1.7) 3 (5)	0.693
**Tumor location** **➢** ** Proximal** **➢** ** Middle** **➢** ** Distal** **➢** ** Diffuse**	28 (46.7) 22 (36.7) 6 (10) 4 (6.7)	17 (28.3) 14 (23.3) 4 (6.7) 4 (6.7)	11 (18.3) 8 (13.3) 2 (3.3) 0 (0)	0.554
**T classification** **➢** ** pT1** **➢** ** pT2** **➢** ** pT3** **➢** ** pT4**	0 (0) 13 (18.3) 19 (31.7) 28 (46.7)	0 (0) 6 (10) 11 (18.3) 22 (36.7)	0 (0) 7 (11.7) 8 (13.3) 6 (10)	0.095
**N classification** **➢** ** N0** **➢** ** N1** **➢** ** N2** **➢** ** N3**	19 (31.7) 11 (18.3) 17 (28.3) 13 (21.7)	10 (16.7) 7 (11.7) 11 (18.3) 11 (18.3)	9 (15) 4 (6.7) 6 (10) 2 (3.3)	0.082
**M classification** **➢** ** M0** **➢** ** M1**	42 (70) 18 (30)	25 (41.7) 14 (23.3)	17 (28.3) 4 (6.7)	0.174
**TNM stage** **➢** ** I + II** **➢** ** III + IV**	22 (36.7) 38 (63.3)	10 (16.7) 29 (48.3)	12 (20) 9 (15)	0.016
**AR Expression** **➢** ** underexpressed** **➢** ** overexpressed**	20 (33.3) 40 (66.7)	5 (8.3) 34 (56.7)	15 (25) 6 (10)	0.000

AURKA, Aurora kinase A; AR, androgen receptor. # The 8th TNM Classification of Malignant Tumors proposed by the AJCC/UICC

**AURKA expression in GC and normal tissues.**

**Figure 1:**
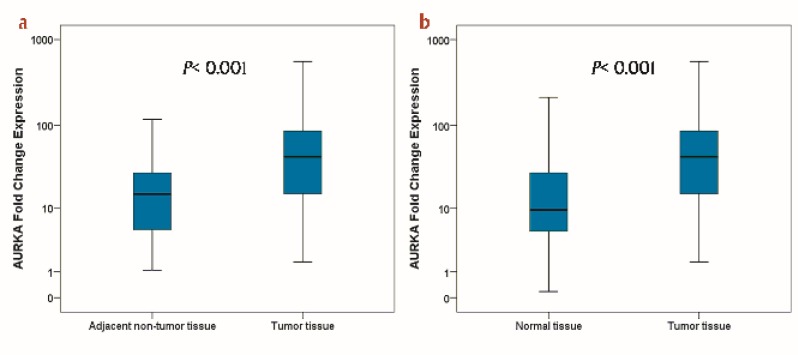
Graphical box-plot expression profile at transcriptome level. Comparing AURKA expression in gastric tumor tissues with (a) adjacent non-tumor and (b) normal tissues. Results are the mean of three independent experiments ± SD (P<0.05).

Quantitative real-time PCR was used to detect the relative AURKA mRNA expression in gastric samples (Figure 1). 

The results showed significantly higher values of AURKA expression in GC tissues compared to adjacent non-tumor tissues and normal tissues (median of fold change expression, 42.58 vs 14.94, *P< *0.001; 42.58 vs 9.49, *p*< 0.001 respectively).

**Correlations between mRNA expression of AURKA and Androgen Receptor.**

**Figure 2:**
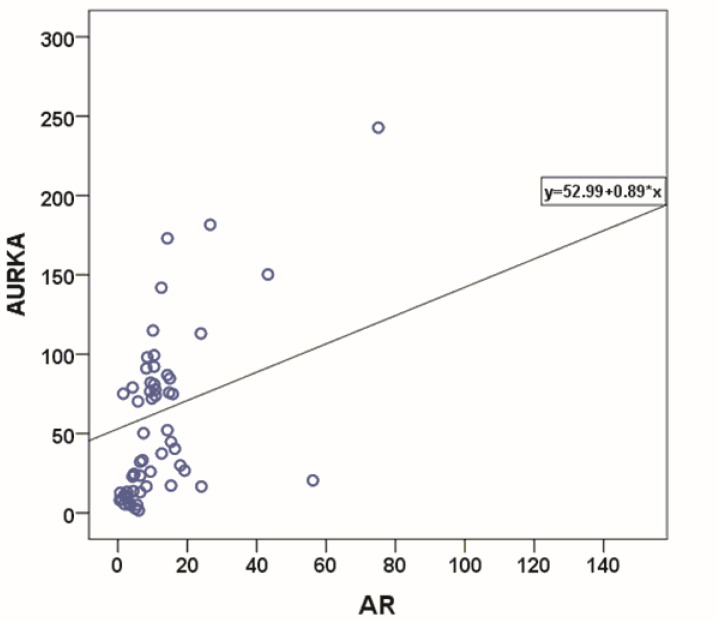
Relationship between AR and AURKA expression in GC samples using Spearman rank test.

Spearman rank test was applied to determine the correlation between expression of AURKA and AR. Almost, strong relationship with a statistically significant correlation coefficient was detected between these two genes expression (r=0.67, *p *<0.001) (Figure 2). 

**AURKA expression correlates with overall survival of gastric cancer patients.**

**Figure 3:**
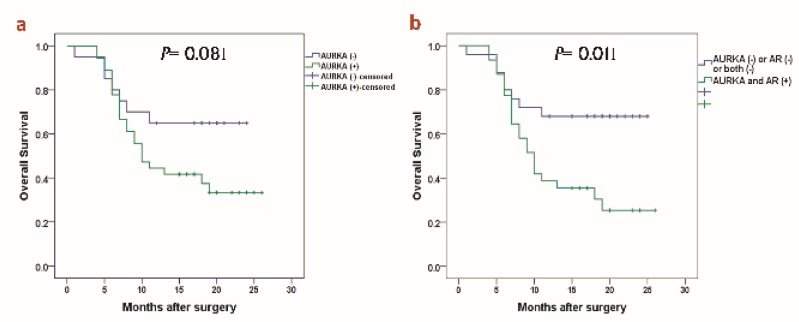
Kaplan-Meier curves of O.S for GC patients according to (a) AURKA expression. (b) O.S for GC patients who simultaneously overexpressed AURKA and AR (log-rank test).

In the present study, GC patients were followed up for 26 months after their surgery. Three patients who failed to contact were lost to follow up. Among patients who overexpressed AURKA, 63.8% and among patients underexpressed AURKA, 35% passed away during this study. However, the correlation between AURKA mRNA expression and O.S of GC patients was not statistically significant using Kaplan-Meier analysis (Figure 3a).

Moreover, we wondered if GC patients who simultaneously overexpressed AURKA and AR genes had lower overall survival than other GC patients (Figure 3b). Interestingly, our data revealed the higher rate of death among these GC patients (70.96%) which was statistically remarkable too.

Furthermore, we measured the prognostic role of clinicopathological characteristics and AURKA expression in GC patients by univariate and multivariate Cox regression analysis (Table 3).

We recently detected that AR gene overexpression associates with poor prognosis of GC patients. In the present study, among all clinicopathological characteristics, T classification, N classification, advanced TNM stages, and simultaneous overexpression of AURKA and AR were significantly correlated with survival of GC patients according to univariate analysis. Lymphovascular invasion was marginally significant (p=0.052). However, multivariate analysis showed that after adjustment with other‌ variables, only TNM stage and simultaneous overexpression of AURKA and AR remained in the model.

**Table 3:** Univariate and multivariate Cox regression analysis of overall survival in patients with gastric cancer.

Variable	Univariate Cox			Multivariate Cox		
	HR	95% CI	*p*	HR	95% CI	*p*
**Sex** **➢ male** **➢ female**	1.000 0.785	0.388-1.591	0.785			
**Age** **(years) median, range** **➢ n < 63** **➢ n ≥ 63**	1.000 1.026	0.493-2.133	0.946	1.000 0.801	0.212-1.776	0.597
**Tumor size (cm)** **➢ n <5** **➢ n ≥ 5**	1.000 2.520	0.968-6.559	0.058			
**Lauren’s classification ** **➢ Intestinal** **➢ Diffuse**	1.000 2.452	0.741-8.112	0. 142			
**Tumor grade** **➢ well differentiated** **➢ moderately** **differentiated** **➢ poorly differentiated**	1.000 1.184 1.337	0.272-5.150 0.296-6.040	0.822 0.705	1.000 1.304 2.465	0.888-5.130 0.957-11.156	0.326 0.087
**Lymphovascular invasion** **➢ No** **➢ Yes**	1.000 2.587	0.990-6.759	**0.052**	1.000 1.176	0.831-5.230	0.104
**perineural invasion** **➢ No** **➢ Yes**	1.000 3.083	0.736-12.916	0.181			
**T classification** **➢ pT1** **➢ pT2** **➢ pT3** **➢ pT4**	1.000 2.133 3.663	0.576-7.900 1.085-12.374	0.257 0.037	1.000 1.788 2.526	0.385-7.297 0.531-12.025	0.244 0.458
**N classification** **➢ N0** **➢ N1** **➢ N2** **➢ N3**	1.000 4.924 7.435 9.801	1.268-19.116 2.080-26.573 2.661-36.104	0.021 0.006 0.001	1.000 2.857 2.287 2.719	0.680-12.002 0.515-10.161 0.582-12.711	0.152 0.277 0.204
**TNM stage** **➢ I + II** **➢ III + IV**	1.000 8.009	2.429-26.411	0.001	1.000 7.671	2.314-25.427	0.001
**AR** **➢ Underexpressed** **➢ Overexpressed**	1.000 4.147	1.582-10.874	0.004	1.000 1.989	0.807-4.626	0.084
**AURKA** **➢ Underexpressed** **➢ Overexpressed**	1.000 1.811	0.808-4.060	0.149			
**AURKA and AR** **➢ Underexpressed** **➢ Overexpressed**	1.000 2.418	1.107-5.280	0.027	1.000 1.668	1.314-3.833	0.042

HR, hazard ratio; CI, confidence interval; AURKA, Aurora kinase A; AR, androgen receptor. # The 8th TNM Classification of Malignant Tumors proposed by the AJCC/UICC

## Discussion

 The aim of this study was to investigate the potential association between AURKA and AR genes expression resulting in GC progression to the end stages. The rationale behind this hypothesis is as follows:

Firstly, various studies have indicated overexpression and amplification of AURKA in GI tumors ^6^^-^^8^. Moreover, it has been well defined that AURKA plays a pivotal role in regulating cell cycle and several oncogenic pathways ^4^^, ^^5^. For instance, a study reported that AURKA could induce cell survival and tumor progression by regulating inhibition of P53 in gastric cancer ^18^. Katsha et al. have shown that AURKA regulates JAK2 expression and phosphorylation to promote STAT3 activity in gastric and esophagus cancers ^19^. Another study in gastric cancer demonstrated that AURKA promotes epithelial-mesenchymal transition through regulating Wnt/β-catenin and PI3K/Akt signaling pathways ^20^.

Secondly, It is well defined that AR, rather than androgen, could function as an oncoprotein by modulating proliferation and metastasis especially in male-predominant tumors ^9^^-^^11^^, ^^21^. Recently, some studies have devoted on indicating the oncogenic role of AR in gastric cancer. In accordance with these new researches, we have demonstrated the implication of AR in GC progression by crosstalk with cell cycle and EMT-related genes such as P53, P21, P27, Cyclin D1, E-cadherin, and β-catenin ^12^^, ^^22^^, ^^23^. 

Furthermore, there is some evidence that indicates the interaction between AURKA and AR in prostate cancer ^14^^-^^16^. They have shown that overexpression of AR in prostate cancer cells could increase AURKA expression ^14^. Another recent study proved this correlation among AR-positive CRPC patients ^16^. They, also, used an AURKA specific inhibitor (alisertib) and indicated that it could significantly reduce the growth of CRPC cells highly expressing AR. Moreover, ChIP-seq analyses have revealed a specific AR binding site in the promoter and in the intronic region of the AURKA gene in prostate cancer ^24^^, ^^25^.

Having said the above, in the current study, we hypothesized that AR and AURKA may interact to promote GC progression and development. We, for the first time, investigated the correlation between the expressions of these two genes among GC patients. It is revealed that among GC patients overexpressing AR, 85% (34/40) had AURKA overexpression too. This result is in consistency with previous studies on prostate cancer ^14^^, ^^16^. We also observed a nearly strong positive correlation between AR and AURKA expression in GC samples using Spearman rank test (r=0.68) which was statistically significant. In consistent with our result, Kivinummi, et al. reported an association with a strong correlation coefficient between the expression of these two genes in prostate cancer patients (r=0.751) ^16^.

Several studies have provided evidence that AURKA overexpression is related to EMT and GC progression ^6^^, ^^16^. Moreover, two different studies have reported the association of the AURKA gene polymorphisms (rs1047972 and rs2273535) with an increased risk of gastric cancer ^7^^, ^^8^. Therefore, we analyzed the correlation between AURKA overexpression and GC patients’ outcome. Although as shown in Table 2, we found that AURKA overexpression is significantly associate with later TNM stages (III +IV), Kaplan-Meier analysis showed no significant correlation with overall survival of GC patients **(**P= 0.081**)**. This result was validated with univariate Cox regression analysis. 

Since the identification of proper markers that precisely predict aggressiveness of gastric cancer could improve the survival of these patients by managing their treatments, we asked if combined evaluation of AR and AURKA expression could introduce a proper prognostic marker. Interestingly, Kaplan-Meier analysis showed that patients who overexpressed both genes remarkably had worse outcome than others.

Furthermore, simultaneous assessment of AURKA and AR genes overexpression, as a single variable, turned out to be an independent unfavorable factor for O.S of GC patients adjusted for other variables using multivariate Cox regression model (HR=1.7, p=0.042). 

## CONCLUSION

 In conclusion, we believe that these findings have clinical value because of the fact that they demonstrate involving of AR and AURKA interaction in GC progression. Moreover, our study provided evidence elucidating a novel promising marker, simultaneous evaluation of AURKA and AR expression, which properly predict prognosis of gastric cancer patients. However, elucidating the exact mechanism of interaction between them warrants further investigations.
